# Automatic Sleep Staging Algorithm Based on Time Attention Mechanism

**DOI:** 10.3389/fnhum.2021.692054

**Published:** 2021-08-17

**Authors:** Li-Xiao Feng, Xin Li, Hong-Yu Wang, Wen-Yin Zheng, Yong-Qing Zhang, Dong-Rui Gao, Man-Qing Wang

**Affiliations:** ^1^Department of Computer Science, Chengdu University of Information Technology, Chengdu, China; ^2^Department of Biological Engineering, University of Electronic Science and Technology of China, Chengdu, China

**Keywords:** Bi-directional gated recurrent unit, conditional random field, class balance strategy, sleep staging, time attention mechanism

## Abstract

The most important part of sleep quality assessment is the automatic classification of sleep stages. Sleep staging is helpful in the diagnosis of sleep-related diseases. This study proposes an automatic sleep staging algorithm based on the time attention mechanism. Time-frequency and non-linear features are extracted from the physiological signals of six channels and then normalized. The time attention mechanism combined with the two-way bi-directional gated recurrent unit (GRU) was used to reduce computing resources and time costs, and the conditional random field (CRF) was used to obtain information between tags. After five-fold cross-validation on the Sleep-EDF dataset, the values of accuracy, WF1, and Kappa were 0.9218, 0.9177, and 0.8751, respectively. After five-fold cross-validation on the our own dataset, the values of accuracy, WF1, and Kappa were 0.9006, 0.8991, and 0.8664, respectively, which is better than the result of the latest algorithm. In the study of sleep staging, the recognition rate of the N1 stage was low, and the imbalance has always been a problem. Therefore, this study introduces a type of balancing strategy. By adopting the proposed strategy, SEN-N1 and ACC of 0.7 and 0.86, respectively, can be achieved. The experimental results show that compared to the latest method, the proposed model can achieve significantly better performance and significantly improve the recognition rate of the N1 period. The performance comparison of different channels shows that even when the EEG channel was not used, considerable accuracy can be obtained.

## 1. Introduction

Sleep is the most important physiological activity of human beings. Poor sleep can endanger the human immune system and threaten people's lives and health. Numerous studies have shown that more and more drivers are face with irreparable consequences due to fatigue driving (Moul et al., 2002; RN2, 2005; Sateia et al., 2017). Individuals with severe sleep-related diseases suffer from sleep fragmentation and apnea during sleep. When they start to enter a deeper stage of sleep, their airways can become blocked and interfere with their normal breathing. This interference forces the body to return to a lighter sleep stage to continue breathing better. People with sleep apnea do not cycle through the normal phases of the sleep cycle. Therefore, analyzing sleep status can understand sleep conditions, design sleep disorder prevention strategies, and protect people's sleep health. In order to get the sleep state throughout the night, different sleep stages must be classified. In other words, to study sleep-related diseases and disorders more profoundly, the accuracy of sleep stage detection must be improved. At present, polysomnography (PSG) has been mainly used clinically for sleep assessment. The PSG needs to record many physiological signals, such as electroencephalogram (EEG), electrocardiogram (ECG), electromyography (EMG), electrooculogram (EOG), pulse oximetry, and respiratory signals. The sleep stage usually includes night wake (Wake), rapid eye movement (REM) stage and non-rapid eye movement (NREM) stage. According to the new standard (Iber et al., 2007; Danker-Hopfe et al., 2010) rules of the American Academy of Sleep Medicine (AASM), the NREM stage can be further divided into N1, N2, and N3 stages. The old standard Rechtschaffen and Kales (R&K) rules divide the N3 stage into S3 and S4 (Rechtschaffen, 1968).

In the traditional sleep staging methods, sleep stages are classified based on monitoring signals, which is time-consuming, laborious, and prone to the subjective influence of sleep experts (Collop, 2008). Therefore, many efforts have been put into developing automatic sleep staging methods. The staging algorithms can be roughly divided into two categories, traditional machine learning-based algorithms and deep learning-based algorithms that use artificial neural networks.

In the early days, automatic sleep staging was performed by extracting features using machine learning-based algorithms. The most common machine learning-based classification methods include decision trees, random forests (Fraiwan et al., 2012), and support vector machines (Koley and Dey, 2012). However, algorithms that combine feature extraction and traditional machine learning generally have certain shortcomings, such as low accuracy, requirements for large-scale training samples, low recognition rate in the N1 period, and ignoring the temporal connection between tags, so their practicability is not high.

With the development of artificial neural networks, deep learning has gradually become popular in the field of sleep staging. Deep learning represents a new research direction in the field of machine learning, and it combines low-level features to form more abstract high-level representation attribute categories or features to discover distributed feature representations of data. In the field of sleep staging, mainly convolutional neural networks (CNNs), recurrent neural networks (RNNs), and their variants have been used. For instance, the variants of CNN are residual networks and graph convolutional networks, and the variants of RNN are gated recurrent unit (GRU) and long-short term memory (LSTM). The combination of CNN and RNN has also been often used in sleep staging research.

However, the application of deep learning to the existing automatic sleep staging algorithms has certain limitations, which can be summarized as follows.

1) The deep learning networks have too many levels, consume much computing resource and time, and are not practical.
2) Most algorithms use RNN to extract the time information of the signal while ignoring the relationships between sleep states.
3) N1 phase is the transitional phase between the Wake phase and the N2 phase, which plays a crucial role in studying the process of falling asleep and sleep regulation. However, owing to a small number of samples in the N1 stage, and it can be easily misjudged as the Wake stage or the N2 stage. The recognition rate of the N1 stage of the existing sleep staging algorithms is below 0.5, and there is also a class imbalance problem (Penzel et al., 2013; Rosenberg and Hout, 2014). Thus, improving the recognition rate of the N1 phase has been important and challenging.

**Figure 1:**
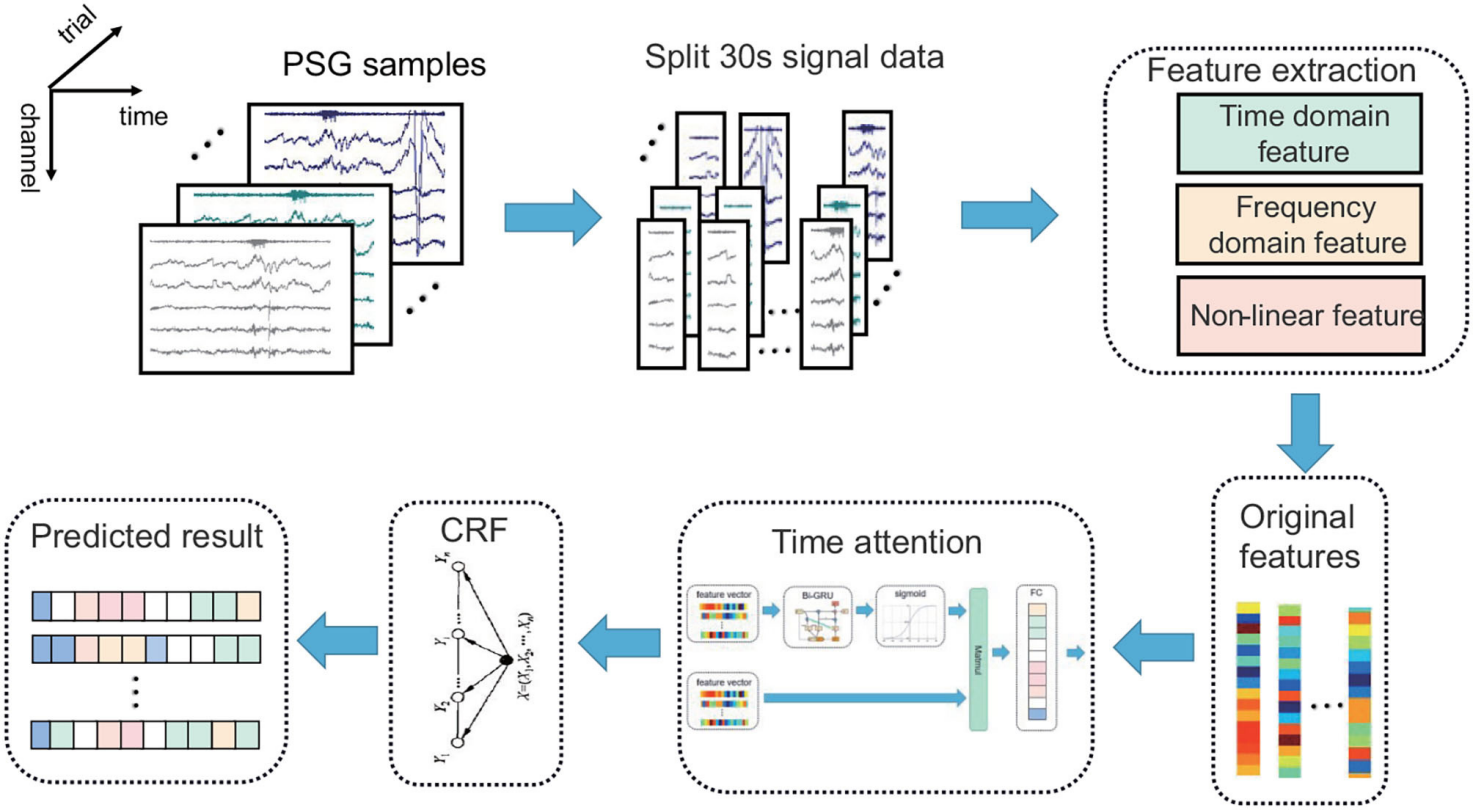
The architecture of the automatic sleep stage classification model based on the time attention mechanism combined with the CRF model. The PSG is divided into 30-s epochs; the original features are obtained via feature extraction, and the prediction results are obtained by the time attention mechanism combined with the CRF model.

To overcome the mentioned limitations, this study proposes an automatic sleep staging algorithm based on the time attention mechanism. The outline of the proposed algorithm is shown in Figure 1. The main steps are feature extraction, temporal attention extraction of temporal features, CRF model extraction of label continuity, and classification.

The main contributions of this research are as follows.

1) Combining the time attention mechanism with Bi-GRU as a model could effectively extract time information and reduce computing resources and time costs.
2) CRF was used to extract the feature information between tags to modify the sleep staging state obtained by the temporal attention model.
3) The class balance strategy was adopted, and the weight between different classes, thus improving the N1 recognition rate.
4) In addition, the classification accuracies of different channels and different channel combinations are compared. The results show that the proposed method can achieve considerable accuracy without using the EEG channels.

The rest of this study is organized as follows. In section Related Work, a detailed description of the related work is presented. In section Materials and Methods, the model architecture and experimental process are described. The results reflecting the proposed model's performance are presented in section Results. In section Discussion, the discussion and future work are presented. Lastly, in section Conclusion, the conclusions are drawn.

## 2. Related work

### 2.1. Sleep Staging

Sleep staging refers to the classification of sleep into five sleep stages formulated by AASM by sleep researchers as the staging standard. The five sleep stages are Wake, N1, N2, N3, and REM. EEG is usually described by its frequency components. The slow waves include the activities that are at the frequency in the range of 0.5–2.0 Hz, where the minimum amplitude of positive and negative peak-to-peak values recorded by the frontal leads is 75 mv, and also δ(0.5–4 Hz), θ(4–8 Hz), α (8–12 Hz), and β(12–35 Hz) waves (Kamran et al., 2019).

In Fraiwan et al. (2012), the classification of sleep stages was conducted by extracting time-frequency features and entropy features using the random forest classifiers. In Liang et al. (2012), multi-scale entropy and autoregressive features were used for sleep staging. In Zhu et al. (2014), time-frequency features were extracted, and the support vector machines were used for the classification of sleep stages. In Hassan and Haque (2015), the empirical model decomposition was used to extract features, and this model was combined with decision trees for the classification of sleep stages. In Hassan and Bhuiyan (2016), the classification of sleep stages was performed using wavelet transform with the adjustable Q factor and random forest classifier.

Recently, deep learning has achieved many significant results in the field of sleep staging. In 2016, Tsinalis et al. (2016) used a two-layer convolutional neural network to perform single-channel EEG automatic sleep staging. Although the manual feature extraction steps were simplified, the accuracy rate was only 71–76%. In 2017, Supratak et al. (2017) combined the CNN model with the LSTM algorithm to introduce residual learning for sequence classification, which could learn the time information of EEG, and achieved the accuracy of 86–82%. However, this model has different recognition capabilities for EEG signals from different channels, so changing the EEG channel has a great impact on the results. In Olesen et al. (2018) used a large-scale multi-channel dataset and adopted a 50-layer convolutional CNN model to obtain an accuracy of 84%, but the proposed CNN model had a large number of layers and consumed too much computing resource. Patanaik et al. (2018) used deep convolutional networks and multi-layer perceptron for sleep staging, and achieved the accuracy of up to 89.8%, and also comprehensively analyzed the sleep characteristics of different people; however, this multi-layer neural network consumes too much computing resource and time.

### 2.2. Bi-Directional Gated Recurrent Unit

GRU is a type of recurrent neural network. Like LSTM, the GRU was initially proposed to solve the problems of long-term memory and gradients of the backpropagation method (Hochreiter and Schmidhuber, 1997). Compared with the LSTM, the GRU can achieve considerable results, and its training process is easier, which can significantly improve the training efficiency and save computing resources and time cost (Cho et al., 2014; Chung et al., 2014).

### 2.3. Time Attention Mechanism

The time attention mechanism assigns weight to each time point in the time series, and the data information of the time point with a greater degree of relative correlation has a higher weight distribution. Namely, the attention mechanism provides different intermediate vector *Ci* using the sigmoid function. The intermediate vector *Ci* contains the influence of the information of each time point of time series *X* on the data prediction at different times, which can save training time. In other words, the time attention mechanism will assign higher weights to epochs with a greater degree of relative relevance, which is conducive to distinguishing long-term continuous staging, while epochs with a lower degree of relative relevance will be assigned lower weights, which will reduce the up and down fluctuations during the staging process, and can also eliminate unimportant information, thus saving the training time.

### 2.4. Conditional Random Field

**Figure 2:**
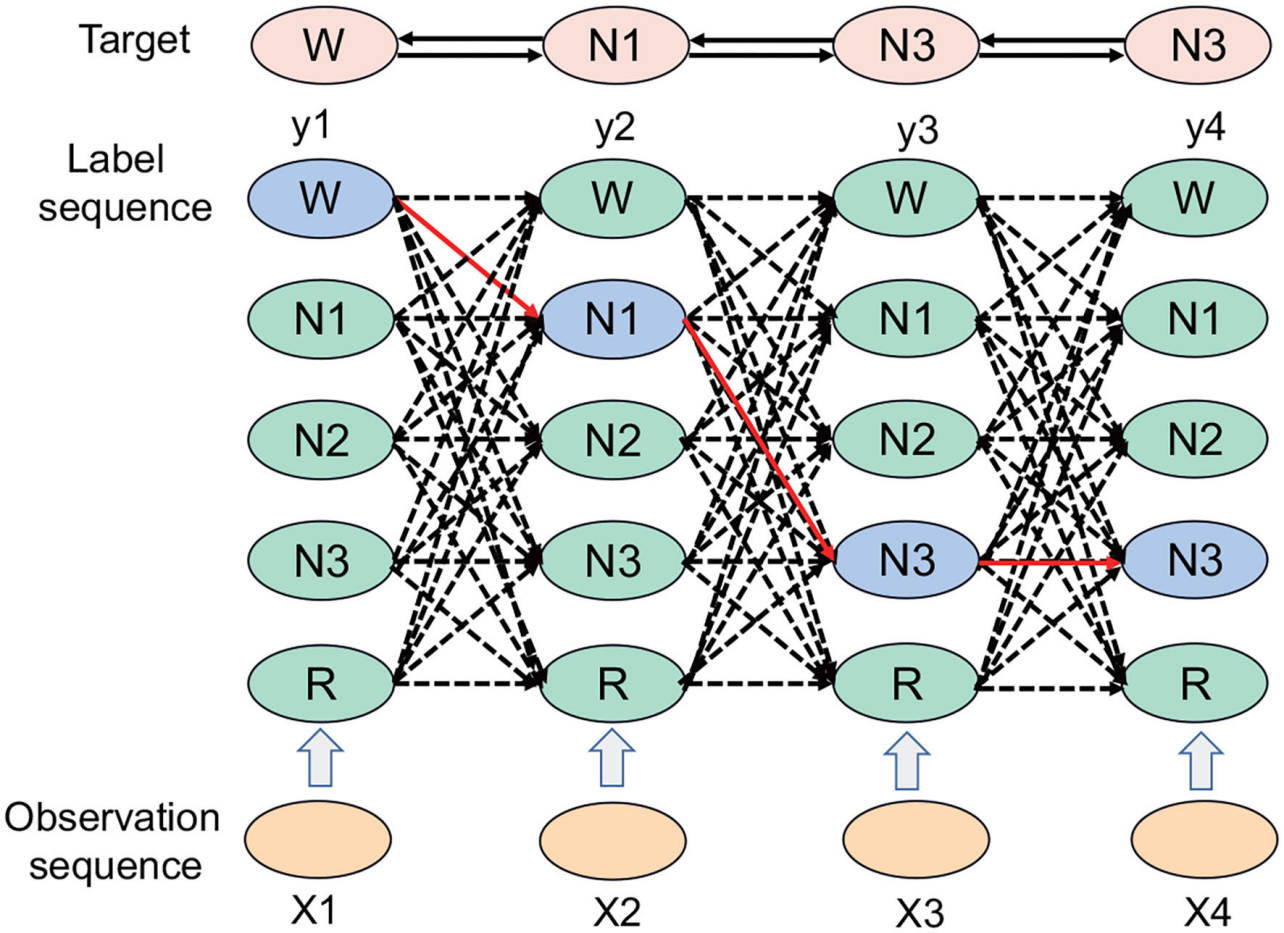
The CRF path selection process. Each column represents a time node. There are five periods: W period, N1 period, N2 period, N3 period, and REM period; the solid black line denotes the final selected path, which includes the W, N1, N3, and N3 periods.

The CRF represents an undirected graph model defined by the hidden Markov process, and *X* denotes the entire observable vector. The CRF separates the associations at the output level so that the model can learn the connection between sequence tags and also takes the path as a unit, considering the path probability. If an input has n frames and the label of each frame has k possibilities, then theoretically, there are *k*^*n*^ different outputs. In Figure 2, each point represents the possibility of a label, the connection between the points represents the association between the labels, and each labeling result corresponds to a complete path in the graph. The correct path is selected from *k*^*n*^ paths, which represents a classification problem of selecting one category from *k*^*n*^ categories.

The CRF represents a machine learning model. In the experiment of the automatic sleep staging algorithm based on the time attention mechanism, the CRF acts as a loss function, which is good for extracting the relationships between the tags. There are certain relationships between tags; for instance, the W period can be the N1 period or the N2 period, but it is unlikely to be the N3 period. The CRF can add the relationship between this type of sequence tag in the sleep state, and correct and overcome the limitations of the time attention mechanism combined with the Bi-GRU.

## 3. Materials and Methods

### 3.1. Datasets

#### 3.1.1. Sleep-EDF Datasetm

Sleep-EDF datasets were used in this study, which are available in the Public PhysioNet Database (Kemp et al., 2000). Sleep-EDF dataset contains recorded signals of 8 healthy males and females, 21–36 years old, who did not take any medication. The dataset consists of two group of subjects, 4 sleep cassette (SC) recording that collected in 1989 during 24 h daily life at the home and 4 sleep telemetry (ST) recording that collected in 1994 during overnight sleep in the hospital from subjects who had mild difficulty falling asleep. The recordings contain horizontal EOG, FpzCz and PzOz EEG, each sampled at 100 Hz. The sc* recordings also contain the submental-EMG envelope, oro-nasal airflow, rectal body temperature and an event marker, all sampled at 1 Hz. The st* recordings contain submental EMG sampled at 100 Hz and an event marker sampled at 1 Hz.

#### 3.1.2. Self-Collected Dataset

Based on clinical considerations and in accordance with the AASM manual, this study completed 83 nights of polysomnography experiments. There are 21 subjects in total, and each subject has a continuous experiment ranging from 1 night to 5 nights. The subjects were all healthy young people, ranging in age from 20 to 24 years old, and the ratio of male to female was 1:0.384. All subjects volunteered to participate in this sleep experiment. Before the experiment, they were required to wash their hair and bath to keep their head clean. The experiment time was mainly from 23:00 on the same day to 07:30 the next day, and the sleep data was >8 h. All subjects ensured good health, no recent medication history, and no strenuous exercise within 1 h before the start of the sleep experiment.

This sleep experiment used a self-designed polysomnography monitor to complete the accurate acquisition and storage of 3-channel EEG, 2-channel EOG, 1-channel EMG, and 1-channel ECG signals in the sleep experiment. The sampling rate is 250 Hz, and the system gain is 24. In addition, the EEG electrodes used for signal acquisition all use gold-plated disc electrodes and are used with gel conductive paste. EOG, EMG and ECG electrodes all use patch electrodes. The entire sleep experiment process was completed on the subject's own dormitory bed, and all electrode placement positions were recommended by AASM. In order to simplify the electrode title, we unified the three EEG channels, namely C4-A1, F4-A1 and O2-A1, simplified to C4, F4, and O2. Then the two EOG channels, namely EOG-R and EOG-L, are simplified into REOG and LEOG.

We perform statistics based on the sleep stage labels classified by doctors, and obtain the number of samples at each stage. The number of data samples of the Wake, N1, N2, N3, and REM stages were 22,726, 4,296, 34,316, 14,910, and 18,372, respectively. The sample size of the N1 stage was the smallest, and the sample size of the N2 stage was the largest. Thus, there was a problem of class imbalance. A total of 94,620 samples were collected.

### 3.2. Data Preprocessing

The data processing steps were as follows:

1) Cutoff frequency design: Use high-pass filter and low-pass filter to remove the noise of each channel, EEG (0.3–35 hz), EOG (0.3–35 hz), EMG (10–100 hz), ECG (0.3–70 hz).
2) Filter design: Choose a Butterworth filter with infinite impulse response IIR to filter the collected data to avoid phase shifts caused by EEG, EOG, EMG, and ECG.
3) According to the AASM staging standard, the filtered data is divided into non-overlapping segments in a time series of 30 s/epoch. And request sleep physicians to stage sleep data according to sleep discrimination criteria and their own experience, and their staging results are used as labels.
4) Trim the data on each night to nine and a half hours; complement data that last less than nine and a half hours with the previous consecutive Wake period; trim data of more than nine and a half hours to keep the number of samples per night consistent.

### 3.3. Feature Extraction

Feature extraction corresponds to the extraction of characteristic patterns of EEG signals in different sleep stages, which is an important step in the automatic classification of different sleep stages. The signal is used as the original sequence, and δ(0.5–4 Hz), θ(4–8 Hz), α(8–13 Hz), and β(13–30 Hz) frequency band are extracted from the original sequence using welch and lomb methods to obtain eight new frequency band. Secondly, the original sequence is regarded as the first-order difference to obtain a new sequence, so together with the original sequences, a total of 10 sequences or frequency band are obtained. By extracting 14 time-domain features from these 10 data sequences or frequency band, plus 12 non-linear features, each channel has 152 features. The extracted features are shown in Table 1.

**Table 1:** Extracted features.

**Symbol**	**Description**	**Feature category**
max	Maximum	Time-domain feature
min	Minimum	Time-domain feature
mean	Mean	Time-domain feature
var	Variance	Time-domain feature
std	Standard deviation	Time-domain feature
per25	25% quantile	Time-domain feature
per75	75% quantile	Time-domain feature
per95	95% quantile	Time-domain feature
skew	Skewness	Time-domain feature
kurt	Kurtosis	Time-domain feature
median	Median	Time-domain feature
overzero	Zero crossing rate	Time-domain feature
hm	Mobility in Hjorth parameters	Time-domain feature
hc	Complexity in Hjorth parameters	Time-domain feature
delta	Delta energy value	Frequency-domain feature
theta	Theta energy value	Frequency-domain feature
alpha	Alpha energy value	Frequency-domain feature
beta	Beta energy value	Frequency-domain feature
permEn	Displacement entropy	Non-linear feature
sampleEn	Sample entropy	Non-linear feature
SvdEn	Singular value decomposition entropy	Non-linear feature

In the studies on the feature extraction of EEG signals (Samet, 2015), the existing features, including frequency-domain features, time-domain features, and non-linear features, were discussed in detail. The original EEG signal is usually preprocessed by a bandpass filter to extract frequency-domain features (Hell, 2010; Pan et al., 2012; Malaekah, 2016). The time-domain features mainly include the maximum value, minimum value, mean value, variance, standard deviation, 25% quantile, 75% quantile, 95% quantile, skewness, kurtosis, median, zero rate, and Hjorth parameter (Hjorth, 1970).

### 3.4. Network Structure

The extracted (T, F) feature vector was input into the Bi-GRU to extract the time series features, and then the sigmoid function was used to assign a high weight to the time series with high correlation. Then multiply the weight matrix obtained above by the original input feature vector, and the result was input into an FC to obtain the CRF input. Among them, T is the number of epochs, and F is the number of features. In particular, given an input $X=\left\{x_{1},\ldots ,x_{N}\right\}\in {\mathbb{R}}^{N\;\times \;d}$ where N is the total number of features, and d is the length of *x*_*i*_,1 ≤ *i* ≤ *N*. The corresponding formula was as follows:

(1) Attention(X,X)=Sigmoid(GRU(X))X.

**Figure 3:**
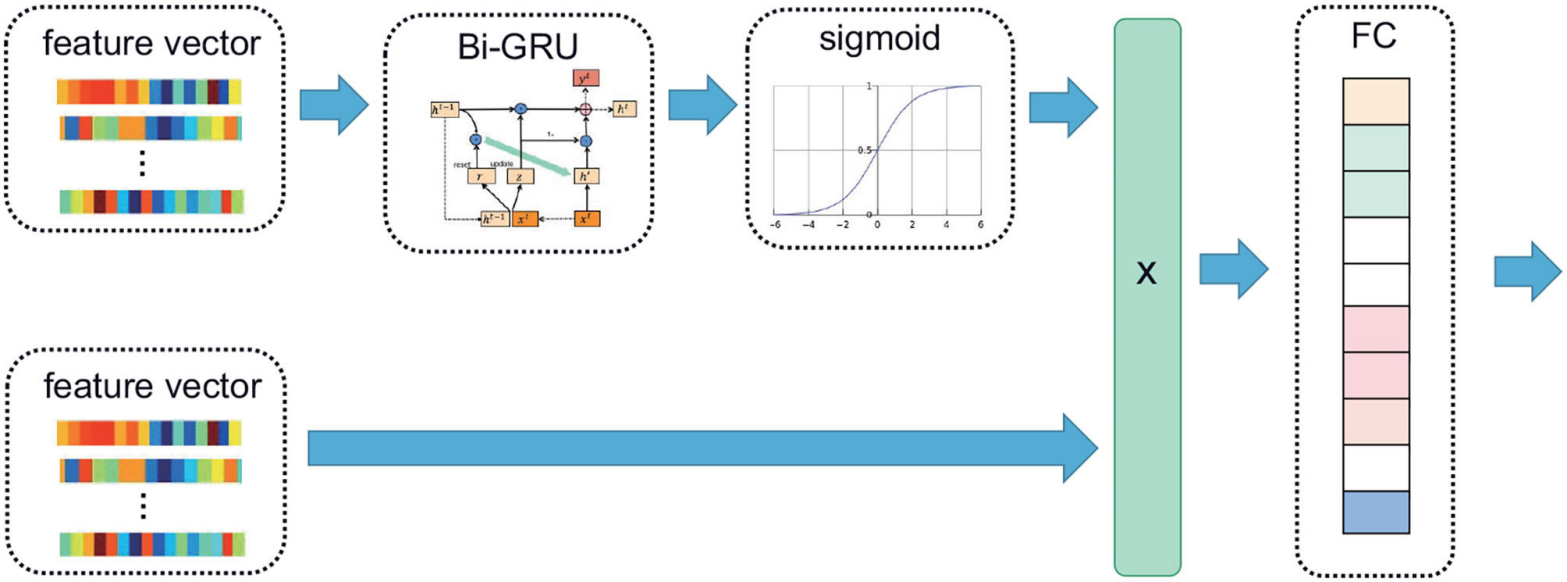
The time attention mechanism. The feature vector extracted from the PSG is input into the Bi-GRU; the time continuity information of the signal is extracted, and high weight is assigned to the time series with high correlation using the sigmoid function. The attention matrix of the same dimension as the feature vector is multiplied with the original feature vector, and the multiplied vector is sent to the linear layer.

Where *X* denoted the input feature vector. Its block diagram is shown in Figure 3.

In the Bi-GRU, the input size was the total number of features, the hidden size was half the number of features, and there was only one hidden layer. There are a total of 912 features, and each channel has 152. They are arranged in order, namely C4, EMG, LEOG, REOG, F4, O2. Different signals can be selected as input in this order to compare the performance of different channels. The number of outputs of the CRF layer was equal to the number of classes; since there were five stages of the classification, the number of outputs was five. The time attention mechanism was trained by using Adam optimizer with a batch size of 1140 examples and a learning rate of 0.0001. The CRF was used as the loss function with a weight decay of 0.01.

### 3.5. Evaluation Indexes

The performance of the proposed model was evaluated regarding the following evaluation metrics: the accuracy (ACC), F1-score (F1), Cohen Kappa (k), and the sensitivity in N1 period (SEN-N1). Given the True Positives (*TP*_*i*_), False Positives (*FP*_*i*_), True Negatives (*TN*_*i*_) and False Negatives (*FN*_*i*_) for the i -th class, the overall accuracy ACC, and F1 are defined as follows.

(2) $$ACC=\frac{\sum_{i=1}^{K}TP_{i}}{M}$$

(3) $$F1=\frac{2\;\times \;{\text{ Precision }}_{i}\;\times \;{\text{ Recall }}_{i}}{{\text{ Precision }}_{i}+{\text{ Recall }}_{i}}$$

where Precision ${\;}_{i}=\frac{TP_{i}}{TP_{i}+FP_{i}}$ and Recall ${\;}_{i}=\frac{TP_{i}}{TP_{i}+FN_{i}}$ . M is the total number of samples and K is the number of classes or sleep stages.

We also used per-class precision (PR), per-class recall (RE) and per-class F1-score (F1) to evaluate each our model. They are calculated as in binary classification by considering one class as the positive class and the other four classes as the negative class.

The Kappa coefficient is used for consistency testing and can also be used to measure classification accuracy. The calculation result is (–1, 1), but usually kappa falls between (0, 1), which can be divided into five groups to indicate different levels of consistency: 0–0.2 means very low consistency, 0.21–0.4 means General consistency, 0.41–0.6 indicates medium consistency, 0.61-0.8 indicates high consistency, 0.81–1 indicates almost complete consistency, the formula is:

(4) $$K=\frac{{\text{P}}_{\text{o}}-{\text{P}}_{\text{e}}}{1-{\text{P}}_{\text{e}}}$$

where Po is accuracy; suppose the number of real samples of each category are a_1_, a_2_, ⋯ , a_m_, and the number of samples predicted for each category are b_1_, b_2_, ⋯ , b_m_, the total number of samples If the number is n, then: ${\text{p}}_{\text{e}}=\frac{{\text{a}}_{1}\;\times \;{\text{b}}_{1}+{\text{a}}_{2}\;\times \;{\text{b}}_{2}+\cdots +{\text{a}}_{\text{m}}\;\times \;{\text{b}}_{\text{m}}}{\text{n }\times \text{ n}}$ .

## 4. Results

### 4.1. Performance of Model

The extracted features were used as input, and the temporal attention model was trained using extracted features from sleep data. To train the model with the whole-night data, the five-fold cross-validation was used to divide the training set and the test set into 83 night-data sets, and the ratio of the training set to the test set was 4:1. Five-fold cross-validation is to divide the data into five parts, take one part of the test each time, and use the remaining part for training. A total of five times are required. The five test sets are all different subject, that is, cross-validation is subject-independent. For example, take out the sleep data test of 17 of the 83 nights, and use the remaining subject for training. Tables 2, 3 show the confusion matrix of the proposed model applied on the Sleep-EDF dataset and our own dataset. The confusion matrix is calculated by adding up all the scoring values of the testing data through the five folds. The table also shows the accuracy, recall and F1 score of each class, as well as the overall accuracy and Kappa.

**Table 2:** Confusion matrix and classification performance of proposed model applied on Sleep-EDF dataset.

		**Wake**	**N1**	**N2**	**N3**	**REM**
	Wake	0.9926	0.0029	0.0029	0.0004	0.0012
	N1	0.1921	0.4177	0.1951	0.0030	0.1921
Expert scored	N2	0.0141	0.0027	0.9478	0.0204	0.0150
	N3	0.0167	0.0000	0.1892	0.7941	0.0000
	REM	0.0167	0.0078	0.2033	0.0000	0.7722
Precision		0.9752	0.8354	0.8365	0.9278	0.8720
Recall		0.9926	0.4177	0.9478	0.7941	0.7722
F1 Score		0.9838	0.5569	0.8887	0.8558	0.8191
Accuracy:0.9218			WF1:0.9177		Kappa:0.8751	

**Table 3:** Confusion matrix and classification performance of proposed model applied on our own dataset.

		**Wake**	**N1**	**N2**	**N3**	**REM**
	Wake	0.9732	0.0170	0.0046	0.0006	0.0046
	N1	0.2085	0.5147	0.1993	0.0103	0.0671
Expert scored	N2	0.0101	0.0222	0.9026	0.0297	0.3530
	N3	0.0057	0.0026	0.1115	0.8775	0.0027
	REM	0.0254	0.0119	0.0474	0.0001	0.9153
Precision		0.9251	0.6088	0.8983	0.9245	0.9114
Recall		0.9732	0.5147	0.9026	0.8775	0.9153
F1 Score		0.9485	0.5578	0.9005	0.9153	0.9134
Accuracy:0.9006			WF1:0.8991		Kappa:0.8664	

As shown in Table 2, after five-fold cross-validation on the Sleep-EDF dataset, the values of accuracy, WF1, and Kappa were 0.9218, 0.9177, and 0.8751, respectively. As shown in Table 3, after five-fold cross-validation on the our own dataset, the values of accuracy, WF1, and Kappa were 0.9006, 0.8991, and 0.8664, respectively. In other words, the Kappa was all above 0.8, thus, the two judgments were almost identical.

The Wake period may be misdiagnosed as the N1 period, which was because, from the beginning of sleep, people gradually fell asleep, while the N1 period was between awake and deep sleep, which was a state of sleep but not sleep. That is, the characteristics of the N1 period are similar to those of the Wake period. Also, the N1 period could be misjudged as the N2 period or the Wake period. The characteristics of the N2 and N3 stages were very obvious, so their recognition rates were above 0.8. The N2 stage may be misjudged as the N3 stage, and the N3 stage may be misjudged as the N2 stage because the N2 and N3 phases had similar structures and were continuous in time. The REM period may be misjudged as the Wake period and N2 period, which was because, The REM period is a transitional state from deep sleep to waking up, and their sleep had certain similarities, but the characteristics of REM itself were obvious.

**Figure 4:**
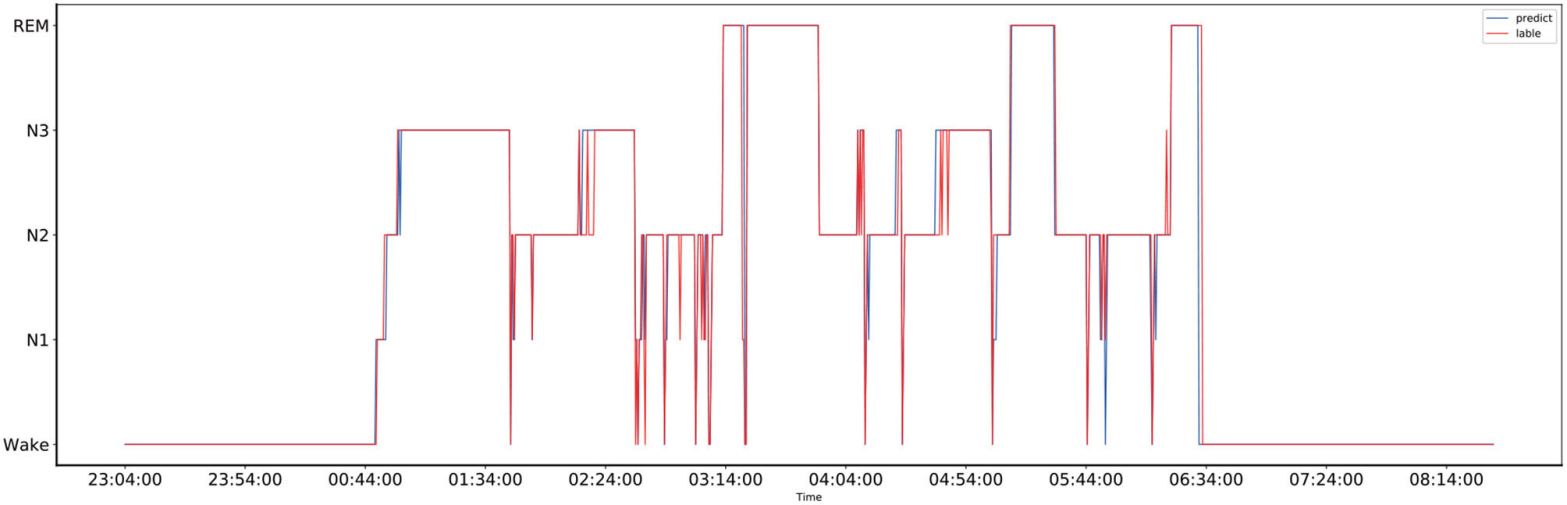
The comparison of the one-night sleep data diagrams, of which one was obtained by manual sleep data staging by a sleep expert and another one was obtained by automatically sleep staging by the proposed model, and they are represented by the red and blue lines, respectively.

**Figure 5:**
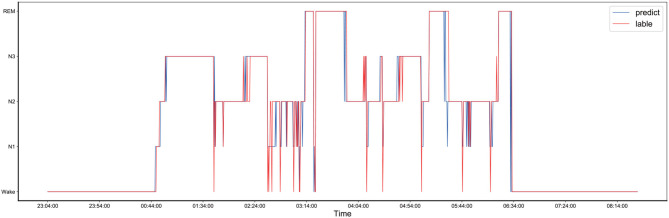
The comparison of the one-night sleep data diagrams, of which one was obtained by manual sleep data staging by a sleep expert and another one was obtained by automatically sleep staging by the proposed model, and they are represented by the red and blue lines, respectively.

The comparison between the manually obtained labels by sleep experts and the labels predicted by the proposed method using the records from the one-night data is presented in Figure 4. The one-night data included a total of 1,140 epochs and lasted nine and a half hours, starting at 23:04 in the evening and ending at 8:14 in the next morning. The sleep data diagram presented in Figure 5 shows that the effective sleep time of the subject was about 6 h, and the subject entered a deep sleep period soon after falling asleep. However, during the sleep, there were many waking times, but after waking, the subject immediately entered a sleep state again. As shown in Figure 4, the Bi-GRU time attention mechanism combined with the CRF model performed well on long time series, but there were fluctuations in the distinction between the N2 and N3 periods, and the N2 period was easily misjudged as the N3 period; also, the N3 period was easily misjudged as the N2 period. The reason was that the model's ability to process continuous time series was too strong, so in the case of sudden jumps, such as the transition from N2 to N3 period, the response was not very sensitive, and it was easy to misjudge the jumping fluctuations as a stable N3-period straight line. For the one-night sleep data, the accuracy and F1 score of the proposed model were 0.956 and 0.96, respectively.

### 4.2. N1 Period Imbalance Problem Solution

Class-imbalance refers to the situation where the number of training examples of different categories in the classification task varies greatly. In the sleep stage, owing to the small number of samples in the N1 period, the recognition rate of the N1 period of the existing sleep staging methods is below 0.5, which causes a class imbalance problem. Therefore, without expanding the N1 period data, it is necessary to introduce a class balance strategy of assigning weights to solve the problem of low model sensitivity to the N1 period. The class balance strategy penalizes stages with a large sample size by adjusting the weights, such as the W stage, which gives the W stage a lower weight and at the same time gives N1 a higher weight.

Using the Hyperband method, which regards each hyperparameter configuration as an arm, and then selects the optimal hyperparameter configuration, the optimal weight parameters were selected. The weights of Wake, N1, N2, N3, and REM periods were set to 1, 6, 1, 2, and 1, respectively, and the confusion matrix and classification performance of proposed model applied on Sleep-EDF dataset and our own dataset after increasing the weight of the N1 state are shown in Tables 4, 5.

**Table 4:** Confusion matrix and classification performance of proposed model applied on Sleep-EDF dataset after increasing the weight of the N1 state.

		**Wake**	**N1**	**N2**	**N3**	**REM**
	Wake	0.9426	0.0364	0.0142	0.0009	0.0059
	N1	0.0504	0.7425	0.1231	0.0243	0.0597
Expert scored	N2	0.0111	0.0522	0.8542	0.0446	0.0379
	N3	0.0183	0.0095	0.1852	0.7822	0.0048
	REM	0.0061	0.1726	0.1889	0.0068	0.6257
Precision		0.9871	0.3455	0.8203	0.8346	0.8030
Recall		0.9426	0.7425	0.8542	0.7822	0.6257
F1 Score		0.9643	0.4716	0.8369	0.8076	0.7033
Accuracy:0.8695			WF1:0.8770		Kappa:0.7974	

**Table 5:** Confusion matrix and classification performance of proposed model applied on our own dataset after increasing the weight of the N1 state.

		**Wake**	**N1**	**N2**	**N3**	**REM**
	Wake	0.8965	0.0928	0.001	0.0001	0.0096
	N1	0.0871	0.7310	0.1067	0.0037	0.0715
Expert scored	N2	0.0036	0.0905	0.8386	0.0192	0.0481
	N3	0.0003	0.0137	0.1593	0.8254	0.0014
	REM	0.0225	0.0832	0.0603	0.0005	0.8336
Precision		0.9571	0.3091	0.8783	0.9477	0.8752
Recall		0.8965	0.7310	0.8386	0.8254	0.8336
F1 Score		0.9258	0.4345	0.8580	0.8823	0.8539
Accuracy:0.8446			WF1:0.8583		Kappa:0.7950	

As shown in Table 4, after five-fold cross-validation on the Sleep-EDF dataset, the values of accuracy, WF1, Kappa, and SEN-N1 were 0.8695, 0.8770, 0.7974, and 0.7425, respectively. As shown in Table 5, after five-fold cross-validation on the our own dataset, the values of accuracy, WF1, Kappa and SEN-N1 were 0.8446, 0.8583, 0.7950, and 0.7310, respectively. In other words, even when the weight of the N1 period was increased while the weights of the other categories were reduced, the accuracy of the proposed model in recognizing the five sleep stages could reach a satisfactory level. The SEN-N1 was higher than 0.7, thus far exceeding the performance of the current sleep staging models. Therefore, after changing the weight, the proposed model solved the problems of class imbalance and low recognition rate.

In order to verify whether the use of the class balance strategy has a significant effect on sleep staging, we performed the paired Wilcoxon Signed Ranks Test on the Sleep-EDF dataset and our own dataset. That is, a statistical test is performed between the results in Tables 2, 4 and Tables 3, 5. It is assumed that there is no significant difference in the results of sleep staging with or without the use of the proposed class balance strategy. However, we obtained *p*-value 0.0036 < 0.05 and 0.0024 < 0.05 for the Sleep-EDF dataset and our own dataset, respectively, after the application of paired Wilcoxon Signed Ranks Test. These results reject the hypothesis and indicate that the proposed class balance strategy has a significant effect on the outcomes.

To provide a fair comparison with the results of the unweighted experiment, the sleep data of the same person at the same night were used to draw a sleep structure diagram, and it is presented in Figure 5. As shown in Figure 5, after the introduction of the class-balance strategy, the fluctuation from N1 to N2 after 5:44 was more apparent than that in the sleep structure diagram without the class-balance strategy. For the one-night sleep data, the accuracy and F1 score of the proposed model were 0.926 and 0.93, respectively.

### 4.3. Comparison of Different Channels

Channels C4, EMG, LEOG, REOG, F4, and O2, and the following channel combination LEOG+REOG, EMG+LEOG+REOG,C4+F4,C4+O2,F4+O2, C4+F4+O2, C4+ EMG, C4+LEOG+REOG and C4+REOG+EMG were used to explore the effects of different channels on the sleep staging results.

The comparison results are presented Table 6, where it can be seen that among the three EEG channels, C4 and F4 contained the most information on the sleep quintuple tasks. The accuracy was above 0.85, the Kappa value was above 0.8, and the performance was good. As more channels contain more sleep information, the results of sleep staging are better than single channel. The experimental results show that if there is only a portable EEG acquisition system, a single-channel EEG can also get a better result. However, the classification performance of the O2 channel was slightly worse; the accuracy was 0.8269, and the Kappa value was lower than 0.8. This could be because the electrode placement was different from the first two channels. The O2 channel was located at the back of the brain and was too far from the eye, so the information on eye movement could be easily missed. Therefore, in the absence of EOG, it is better to use C4 and F4 than the O2 channel. The information of the two EOG channels was very similar; the accuracy was about 0.8, and the Kappa value was above 0.7, but the overall effect of the REOG data on sleep staging was better than that of the LEOG data. This proves that the EOG data has a significant influence on sleep staging. The EOG data contain much sleep staging-related information. Thus, when it is inconvenient to collect the EEG data, it is also a good choice to collect the EOG data to conduct the sleep staging. As shown in Table 6, when the EMG channel was used for sleep staging, the result was not very good; namely, the recognition rates of the N1, N3, and REM stages were very low, but the recognition rates of the Wake and N2 stages were good, reaching the value of above 0.8. These results have proven the EMG-channel data contains many features of the Wake and N2 stages, but the other stages could be difficultly distinguished, so using only EMG data for sleep staging is not recommended, and these data should be combined with the data of the other channels.

**Table 6:** The experimental results for different channels and channel combinations.

**Channel**	**Accuracy**	**WF1**	**Kappa**	**Sensitivity**				
				**Wake**	**N1**	**N2**	**N3**	**REM**
C4	0.859	0.84	0.81	0.96	0.4	0.9	0.87	0.81
F4	0.855	0.84	0.8	0.9	0.4	0.91	0.87	0.86
O2	0.827	0.81	0.77	0.94	0.2	0.83	0.86	0.83
LEOG	0.798	0.78	0.72	0.95	0	0.86	0.75	0.71
REOG	0.809	0.79	0.74	0.92	0	0.8	0.89	0.8
EMG	0.554	0.47	0.35	0.87	0	0.83	0	0.23
LEOG+REOG	0.832	0.82	0.77	0.92	0.11	0.83	0.85	0.86
LEOG+REOG+EMG	0.852	0.85	0.8	0.96	0.3	0.88	0.85	0.78
C4+EMG	0.877	0.87	0.83	0.97	0.41	0.91	0.76	0.89
C4+LEOG+REOG	0.879	0.87	0.83	0.93	0.35	0.87	0.85	0.92
C4+REOG+EMG	0.876	0.87	0.82	0.92	0.28	0.88	0.86	0.91
C4+O2	0.861	0.84	0.80	0.95	0.11	0.90	0.87	0.85
C4+F4	0.864	0.85	0.81	0.95	0.11	0.90	0.89	0.85
F4+O2	0.865	0.85	0.81	0.96	0.12	0.90	0.87	0.85
C4+F4+O2	0.872	0.86	0.82	0.96	0.13	0.91	0.89	0.86
ALL	0.901	0.899	0.866	0.97	0.52	0.90	0.88	0.92

The experiment with different channel combinations showed that the combination of two EOG channels could achieve an accuracy of 0.832 in the sleep staging task, and the Kappa value was 0.77. Compared with the single-channel EOG, the sleep staging result of the two-channel EOG was better, which proves that information of the two EOG channels can compensate for each other. In addition, this proves that using EOG data for sleep staging is a good choice when it is inconvenient to collect EEG data. The results showed that the combination of two EOG channels and one EMG channel was the best choice when there were no EEG data available for sleep staging; the accuracy, WF1, and Kappa were 0.852, 0.85, and 0.8, respectively, which was completely comparable to the result of the single-channel EEG data. Hence, the results could meet the performance requirements of sleep staging. When the two EOG channels were used, the accuracy increased by 0.2, thus proving that the EMG channel contains information that EOG cannot distinguish. When the combination of C4 and EMG channels was used in sleep staging, the accuracy, WF1, and Kappa were 0.877, 0.87, and 0.83, respectively. Compared with a single C4 channel, the accuracy improved by 0.2, which proves that the EMG channel data are beneficial to the sleep staging results. In the multi-channel combination experiment, the combined effect of C4 and two EOGs was the second-best effect, the first one after that of the combination of all channels; the accuracy, WF1, and Kappa values were 0.891, 0.89, and 0.85, respectively. Accordingly, when the objective is to reduce the number of channels while obtaining good staging results, channel combinations C4+EMG, C4+REOG+LEOG and C4+REOG+EMG can be used. In the three EEG permutations and combinations, the sleep results are similar, all around 0.86. Prove that the information of the three EEG channels are mostly overlapped. Naturally, the effect of six-channel data was the best; the accuracy rate was 0.901, WF1 was 0.899, and Kappa was 0.866. This was because the information contained in the six channels was more comprehensive.

Table 7 shows the *p*-value statistical testing between different channels. The part in bold is *p* > 0.05, which means there is no significant difference. Since the difference d in the data of the paired samples does not obey the normal distribution, we use the wilcoxon signed ranks test of the paired samples for data analysis. This method can be used for statistical analysis of non-normal distribution data. This article uses paired Wilcoxon Signed Ranks Test to perform statistical analysis on different channels to see whether the results of different channel combinations are statistically different from the results of full channel combinations. Assuming that the results of different channel combinations are not significantly different from the results of the full channel combination, the paired wilcoxon signed ranks test is performed using the combination of C4 and the two EOG channels and the full channel combination, and the *p*-value is 0.0182 < 0.05. The null hypothesis is rejected, that is, C4 and two The combined result of the EOG channel is significantly different from the result of the full channel combination. The paired wilcoxon signed ranks test was performed sequentially using different channel combinations and full channel combinations, and all *p*-values were less than 0.05, proving that the results of different channel combinations were significantly different from the results of full channel combinations. The part in bold in Table 7 is the part with *p* > 0.05, that is, the channel with no significant difference. It can be seen from Table 7 that there is no significant difference between C4 and F4 and can be substituted for each other. And the two channels contain information redundancy. And the staging results of C4 and F4 are not significantly different from LEOG+REOG+EMG. O2 can be replaced by LEOG+REOG. The two EOG channels can also be substituted for each other because they are not significantly different. There is no significant difference between C4+LEOG+REOG and other C4 combination channels, the same is true for C4+REOG+EMG. However, O2 is significantly different from C4 and F4. Prove that O2 may contain information that C4 or F4 does not.

**Table 7:** *P*-value of statistical test of different channels.

***p*** **-Value**	**C4**	**F4**	**O2**	**LEOG**	**REOG**	**EMG**	**LEOG+** ** REOG**
C4							
F4	**0.26**						
O2	0.0007	0.0008					
LEOG	0.0028	0.0021	0.0193				
REOG	0.0003	0.0002	0.0012	**0.5365**			
EMG	0.0002	0.0002	0.0002	0.0005	0.0003		
LEOG+REOG	0.0061	0.0448	**0.1128**	0.0061	0.0081	0.0003	
LEOG+REOG+EMG	**0.2582**	**0.1146**	0.0005	0.0024	0.0001	0.0001	0.0014
C4+EMG	0.0038	0.0465	0.0001	0.0008	0.0005	0.0002	0.0032
C4+LEOG+REOG	0.0029	0.0042	0.0008	0.0022	0.0002	0.0001	0.0086
C4+REOG+EMG	0.0017	0.0009	0.0004	0.0007	0.0002	0.0001	0.0007
C4+O2	0.0411	0.0001	0.0003	0.0008	0.0001	0.0001	0.0041
C4+F4	**0.2712**	0.0003	0.0006	0.0006	0.0004	0.0001	0.0072
F4+O2	0.0263	0.0032	0.0002	0.0013	0.0008	0.0001	0.0027
C4+F4+O2	0.0203	0.0001	0.0005	0.0008	0.0003	0.0002	0.0024
ALL	0.0081	0.0004	0.0004	0.0006	0.0001	0.0001	0.0017
**LEOG+RE** **OG+EMG**	**C4+EMG**	**C4+LEOG** **+REOG**	**C4+RE** **OG+EMG**	**C4+O2**	**C4+F4**	**F4+O2**	**C4+F4** **+O2**
0.0024							
0.0056	**0.1594**						
0.0021	**0.3205**	**0.6555**					
0.0004	**0.2048**	**0.0856**	0.0328				
0.0023	**0.0584**	**0.0595**	0.0276	0.0133			
0.0045	**0.1441**	**0.0617**	0.0451	**0.7281**	0.0165		
0.0008	**0.3321**	**0.1136**	**0.1411**	**0.2829**	0.0139	**0.2531**	
0.0021	0.0019	0.0313	0.0177	0.0071	0.0022	0.0042	0.0042

### 4.4. Comparison of Different Models

Table 8 compares the performance of our proposed method for sleep stage classification using the Sleep-EDF dataset with recent state-of-the-art works in terms of overall accuracy and Cohen's kappa. The highest accuracy, SEN-N1 and Cohen's kappa are highlighted in bold, which show the performance of our proposed method.

**Table 8:** Performance comparison of proposed algorithm with state-of-the-art studies in 5-class sleep staging on sleep-EDF dataset.

**Reference**	**Model**	**Accuracy**	**Kappa**	**SEN-N1**	***p*** **-Value**
Sharma et al. (2017)	DESA	0.911	0.861	0.2285	0.032
Yildirim et al. (2019)	CNN	0.9122	0.868	0.3974	0.018
Hassan and Haque (2015)	Bagging	0.907	0.855	0.4702	0.0185
Hassan and Bhuiyan (2016)	TQWT +RF	0.915	0.865	0.3742	0.0046
Zhu et al. (2014)	DVG +SVM	0.889	0.830	0.3974	0.0069
Zhou et al. (2020)	RF+LGB	0.912	0.865	0.4205	0.008
Hsu (2013)	FNN+PNN	0.872	0.820	0.3874	0.021
Yu-Liang et al. (2013)	Recurrent Neural	0.903	0.82	0.367	0.0051
Dong et al. (2018)	MLP+LSTM	0.864	0.81	0.3416	0.0341
Sun et al. (2020)	CNN+LSTM	0.872	0.82	0.3656	0.0126
Our model	Time attention	**0.9218**	**0.8751**	0.4177	-
	Class balance+Time attention	0.8695	0.7974	**0.7425**	0.0036

The results show that the evaluation indicators (including ACC and Kappa) obtained by the proposed model are competitive with the state-of-the-art results, and the proposed method significantly improves SEN-N1. Among them, the accuracy rate of the time attention model, Kappa and SEN-N1 can reach 0.9218, 0.8751, and 0.4177, respectively. Using class balance strategy combined with time attention model accuracy, Kappa and SEN-N1 can reach 0.8695, 0.7974, and 0.7425, respectively.

Our own dataset were used set to compare the proposed method with the other methods, and the results are shown in Table 9. The part in bold is the best result of this column. The latest proposed methods were used for the comparison. In Dong et al. (2018), the model used by the author is common MLP+LSTM, using single-channel EEG as input, and an accuracy of 85% was obtained. The latest method proposed in 2020 is presented in Sun et al. (2020). The common CNN+LSTM model was used to obtain an accuracy of 86%, which has been very representative. As shown in Table 9, the accuracy of the proposed method was 0.8934, the Kappa value was 0.86, and good sensitivity was obtained in each stage. Also, the proposed method achieved better results than other methods. After adding the class imbalance strategy, the accuracy rate could also compete with the methods proposed in Dong et al. (2018) and Sun et al. (2020), and the N1 recognition rate was significantly improved.

**Table 9:** Results comparison of different methods using our own dataset.

**Reference**	**Model**	**Accuracy**	**Kappa**	**SEN-N1**	***p*** **-Value**
Sharma et al. (2017)	DESA	0.9001	0.85	0.237	0.024
Yildirim et al. (2019)	CNN	0.8941	0.854	0.3843	0.0013
Hassan and Haque (2015)	Bagging	0.898	0.852	0.4962	0.0066
Hassan and Bhuiyan (2016)	TQWT +RF	0.8993	0.862	0.3851	0.0007
Zhu et al. (2014)	DVG +SVM	0.872	0.824	0.4122	0.013
Zhou et al. (2020)	RF+LGB	0.8974	0.861	0.4458	0.0034
Hsu (2013)	FNN+PNN	0.866	0.814	0.4037	0.039
Yu-Liang et al. (2013)	Recurrent Neural	0.889	0.808	0.346	0.0184
Dong et al. (2018)	MLP+LSTM	0.850	0.80	0.31	0.0261
Sun et al. (2020)	CNN+LSTM	0.861	0.81	0.33	0.0359
Our model	Time attention	**0.9006**	**0.87**	0.52	-
	Class balance+Time attention	0.845	0.795	**0.73**	0.0024

In addition, in Tables 8, 9, we performed statistical tests on all comparison algorithms and our proposed algorithm, and all *p*-values were less than 0.05, which proved that our algorithm was significantly different from the comparison algorithm.

## 5. Discussion

In this study, an automatic sleep staging algorithm based on the time attention mechanism combined with Bi-GRU is proposed. The features, including time and spectrum factors, are extracted from the physiological signals of the PSG channel and then normalized. The time attention mechanism combined with the two-way GRU is used to reduce computing resources and time costs, and the CRF is used to obtain the relationships between tags.

It should be noted that the sleep state is continuous in time, but most of the automatic sleep staging results are prone to step fluctuations. For instance, in the continuous and stable N3 stage, the intermediate result can be misjudged as the REM stage, resulting in significant changes in the sleep structure diagram. Previous studies mostly use machine learning models. For example, (Hsu, 2013; Yu-Liang et al., 2013; Zhu et al., 2014; Hassan and Haque, 2015; Hassan and Bhuiyan, 2016; Sharma et al., 2017; Yildirim et al., 2019; Zhou et al., 2020) only analyzes the characteristics of a single sleep state and disrupts training between epochs, cutting off the continuity between tags. Dong et al. (2018) uses MLP to extract features, LSTM to extract time series information, and (Sun et al., 2020) uses CNN combined with LSTM to extract features and time series information. However, LSTM can only extract the temporal context information of a short sequence, and cannot do anything about a long sequence, especially the time sequence information of a sleep state all night. The proposed model has been compared with the 19-layer 1D-CNN model of Yildirim et al. (2019), whose accuracy rate is above 0.9, and which is suitable for people who both fall asleep normally and have difficulty falling asleep. However, the CNN used in Yildirim et al. (2019) can extract less time information, has a large number of layers and strong jumps, and ignores time continuity. In contrast, the time attention introduced in this study assigns higher weights to epochs with a relatively greater degree of relevance, which is conducive to distinguishing long-term continuous staging, while epochs with a relatively smaller degree of relevance are assigned with low weights, which can reduce the fluctuations in sleep staging, especially step fluctuations, and can also eliminate unimportant information, thus saving the training time. The CRF can extract the relationships between tags because the time attention mechanism assigns weights only according to the degree of association of PSG data, ignoring the relationships between tags, so the CRF can overcome the shortcomings of the time attention mechanism.

The proposed method has been verified by the experiments, which were divided into three parts. In the first part, the time attention mechanism combined with the CRF model was used. After five-fold cross-validation on the Sleep-EDF dataset, the values of accuracy, WF1, and Kappa were 0.9218, 0.9177, and 0.8751, respectively. After five-fold cross-validation on the our own dataset, the values of accuracy, WF1, and Kappa were 0.9006, 0.8991, and 0.8664, respectively. In other words, the accuracy of the proposed model in recognition of five sleep stages was above 0.9, and the variance was only 0.68. The results are better than previous sleep staging studies.

As for the research on the attention mechanism, Phan (Phan et al., 2019) combined the attention mechanism with CNN and LSTM, which reduced time consumption to a certain extent, and achieved an accuracy of 0.87. However, the network structure was complex, and the recognition rate of the N1 period was low. In Qu et al. (2020) used CNN to extract features and combined the attention mechanism and residual neural network for sleep staging; they obtained an accuracy of more than 0.84 and solved the problem of a large number of layers of deep learning-based methods. However, the accuracy rate was low, the network structure was complex, and the staging results fluctuated significantly. In contrast, the model proposed in this study has a simple structure and can effectively solve the problem of strong instability of sleep staging results. The experimental results show that the performance of the proposed model is significantly better than that of the latest method (Qu et al., 2020), achieving small variance and strong stability.

In the second part of the experiments, the class balance strategy was introduced to solve the problem of low sensitivity in the N1 period. Namely, since the N1 phase is the transitional phase between the Wake phase and the N2 phase, it plays a very important role in studying the process of falling asleep and sleep regulation. However, due to a relatively small number of data samples of the N1 stage, this stage can be easily misjudged as the Wake or N2 stage, and the recognition rate of the N1 stage of the current sleep staging methods is below 0.5, which results in a class imbalance problem. Previous studies have not considered the problem of class imbalance in sleep state, which has led to the low recognition rate of N1 stage, such as Yu-Liang et al. (2013). Accordingly, improving the recognition rate of the N1 phase has always been an important and challenging task. For instance, although in most related studies, including Sors (Sors et al., 2018) and Stephansen (Stephansen et al., 2018), the accuracy rate of 87% was achieved, the recognition rate of N1 was too low, and the highest accuracy rate of the N1 stage was only 0.58. Therefore, to solve the problem of low sensitivity in the N1 period without expanding the data of the N1 period, this study introduces a class balance strategy of assigning weights. After the introduction of the class balance strategy, the recognition rate of the N1 phase can reach a value of more than 0.7, and the accuracy rate can be higher than 0.86, which can compete with the current methods and significantly improve the recognition rate of the N1 phase.

In the third part of the experiments, different channels and channel combinations, including C4, EMG, LEOG, REOG, F4, O2, LEOG+REOG, EMG+LEOG+REOG, C4+F4,C4+O2,F4+O2,C4+F4+O2, C4+EMG, C4+LEOG+REOG and C4+REOG+EMG, were used to explore their effects on the sleep staging results. The results showed that among the three EEG channels, the C4 channel performed the best in recognition of five sleep stages, achieving an accuracy rate of more than 0.85. The accuracy of the two EOG channels was about 0.8. The results of the experiments with different channel combinations showed that the combination of two EOG channels provided better results in sleep staging than a single EOG channel. Without using the EEG data, the accuracy of the combination of two EOG channels and one EMG channel could reach a value of 0.852, which was completely comparable to the results of a single EEG channel, and the results could also meet the requirements of sleep staging. The combination of C4 and EMG channels achieved a sleep staging accuracy of 0.877. The accuracy of the combination of C4 and two EOG channels was 0.891. Thus, if the main objective is to reduce the number of channels while achieving good sleep staging results, the channel combination of C4+EMG,C4+REOG+EMG or C4+LEOG+REOG can be used.

However, compared with previous studies, the drawbacks and limitations of the proposed algorithm lie in two points. First, the experiment uses 6-channel data in polysomnography, which has a large amount of data and is not as fast as single-channel sleep staging. Second, there are too many experimental features, it takes too much time to extract the features, and there is no screening and comparison of features. Thus, this article analyzes the impact of different channels on staging results only from the perspective of channel comparison. In the future, we can make the following further improvements to make it more effective. First of all, we can reduce the number of channels and ensure that results will not decrease. Second, we can filter the extracted features by feature selection and compare feature importance. Finally, we can develop a better sleep staging model without using EEG data.

## 6. Conclusion

This study proposes an automatic sleep staging method based on the time attention mechanism. The time attention mechanism combined with the two-way GRU was used to reduce computing resources and time costs, and the CRF was used to obtain the relationships between tags. The proposed method iswas verified by the experiments, which can be divided into three parts. In the first part, the time attention mechanism was combined with the CRF model. After five-fold cross-validation on the Sleep-EDF dataset, the values of accuracy, WF1, and Kappa were 0.9218, 0.9177, and 0.8751, respectively. After five-fold cross-validation on the our own dataset, the values of accuracy, WF1, and Kappa were 0.9006, 0.8991, and 0.8664, respectively, which is better than the result of the latest algorithm; the proposed method also has strong stability. In the second part, without expanding the data of the N1 period, the class balance strategy of assigning weights is introduced to solve the problem of low sensitivity in the N1 period. After the introduction of the class balance strategy, the recognition rate of the N1 phase is increased to more than 70%, and the accuracy rate is improved to more than 0.86, which can compete with the results of the current related methods and also significantly improve the recognition rate of the N1 phase. The third part compares different channels and channel combinations. The results show that when the main aim is to used only one channel for sleep staging, the C4 channel should be used; and when the objective is to reduce the number of channels and obtain a good staging result simultaneously, then the channel combination of C4+EMG, C4+REOG+EMG, or C4+REOG+LEOG should be used. In addition, when EEG data cannot be used, EOG and EMG data, if utilized, may also contribute to model performance for excellent sleep staging.

## Data Availability Statement

The raw data supporting the conclusions of this article will be made available by the authors, without undue reservation.

## Ethics Statement

The studies involving human participants were reviewed and approved by National key Laboratory of Human Factor Engineering. Written informed consent for participation was not required for this study in accordance with the national legislation and the institutional requirements. Written informed consent was obtained from the individual(s) for the publication of any potentially identifiable images or data included in this article.

## Author Contributions

L-XF and XL: conceptualization and writing—review and editing. L-XF: methodology, investigation, data curation, and writing—original draft preparation. L-XF, H-YW, and W-YZ: validation. XL: formal analysis and resources. Y-QZ: visualization. M-QW: supervision. H-YW: project administration. D-RG: funding acquisition. All authors have read and approved the final draft of the manuscript.

## Conflict of Interest

The authors declare that the research was conducted in the absence of any commercial or financial relationships that could be construed as a potential conflict of interest.

## Publisher's Note

All claims expressed in this article are solely those of the authors and do not necessarily represent those of their affiliated organizations, or those of the publisher, the editors and the reviewers. Any product that may be evaluated in this article, or claim that may be made by its manufacturer, is not guaranteed or endorsed by the publisher.
